# Interaction and Engagement with an Anxiety Management App: Analysis Using Large-Scale Behavioral Data

**DOI:** 10.2196/mental.9235

**Published:** 2018-10-01

**Authors:** Paul Matthews, Phil Topham, Praminda Caleb-Solly

**Affiliations:** 1 Data Science Group, Computer Science Research Centre Department of Computer Science and Creative Technologies University of the West of England Bristol United Kingdom; 2 University of the West of England Bristol United Kingdom

**Keywords:** anxiety, mobile phone, eMental health, mHealth

## Abstract

**Background:**

SAM (Self-help for Anxiety Management) is a mobile phone app that provides self-help for anxiety management. Launched in 2013, the app has achieved over one million downloads on the iOS and Android platform app stores. Key features of the app are anxiety monitoring, self-help techniques, and social support via a mobile forum (“the Social Cloud”). This paper presents unique insights into eMental health app usage patterns and explores user behaviors and usage of self-help techniques.

**Objective:**

The objective of our study was to investigate behavioral engagement and to establish discernible usage patterns of the app linked to the features of anxiety monitoring, ratings of self-help techniques, and social participation.

**Methods:**

We use data mining techniques on aggregate data obtained from 105,380 registered users of the app’s cloud services.

**Results:**

Engagement generally conformed to common mobile participation patterns with an inverted pyramid or “funnel” of engagement of increasing intensity. We further identified 4 distinct groups of behavioral engagement differentiated by levels of activity in anxiety monitoring and social feature usage. Anxiety levels among all monitoring users were markedly reduced in the first few days of usage with some bounce back effect thereafter. A small group of users demonstrated long-term anxiety reduction (using a robust measure), typically monitored for 12-110 days, with 10-30 discrete updates and showed low levels of social participation.

**Conclusions:**

The data supported our expectation of different usage patterns, given flexible user journeys, and varying commitment in an unstructured mobile phone usage setting. We nevertheless show an aggregate trend of reduction in self-reported anxiety across all minimally-engaged users, while noting that due to the anonymized dataset, we did not have information on users also enrolled in therapy or other intervention while using the app. We find several commonalities between these app-based behavioral patterns and traditional therapy engagement.

## Introduction

### Background

Anxiety is one of the most common mental health problems; in 2013, there were 8.2 million cases of diagnosed anxiety disorders reported in United Kingdom [1]. The cost of anxiety—treatment, health care, and indirect costs such as loss of employment and productivity—was estimated at €11.6 billion [2]. The current demands on mental health services are considerable [3] and at the same time, there may be a lack of help seeking among young people [4,5]. Digital self-help and education tools are seen as possible ways to help alleviate both demand and lack of support seeking and have shown potential to be effective in anxiety reduction [6,7]. The development of SAM (Self-help for Anxiety Management) was driven by a desire to produce a generic, flexible tool for anxiety self-help that provided ease of access and embodied high standards of usability. A report on the development, structure, and functions of the app is available [8]. Although there has been significant uptake, 1,007,469 downloads users in over 100 countries with an average of 40,000 regular users each month as of October 2017, it is important to understand how users are engaging with it, what features are most used, and whether logs of usage and self-reporting measures can provide insights as a first step in evaluating its therapeutic impact. In general, there has been insufficient work on mHealth app engagement and its associations with intended outcomes [9].

This paper reports on the analysis of user data and its therapeutic implications from the first 3 years of SAM’s availability to a global population of users. In this introduction, we will first position this study in terms of approaches to understanding engagement and present related work on behavioral engagement with mHealth apps. Next, we present the overall design philosophy and main features of the app. Based on these reference points, we will then outline our aims for the research.

### Approaches to Engagement

Engagement can be seen to be constituted as the relationship between a consumer and an individual product or service. A rounded view should incorporate emotional, usability, and behavioral factors [10]. Behavioral engagement can be defined in terms of users’ interactions with different app functions and features, both quantitative and longitudinal.

Although our qualitative impact data (eg, from user reviews) provide evidence of usability and emotional engagement and will be the subject of future investigations, this study focuses on behavioral engagement through analysis of app interaction data over time.

### Related Work

We know of no previous work that has looked at user engagement specifically with eMental Health tools. Previous similar work focusing on behavioral aspects of engagement with other kinds of service has looked at recognizable subgroups of users, engagement periods, and correlates of engagement in-app user populations. In their data mining investigation of over 12 million users of a weight loss app, Serrano et al [11] identified the following 3 main subgroups based on the number of times participants weighed in and the number of food days logged: occasional users, basic users, and power users. Power users (1%; 35,649/324,649 sample) showed successful weight loss in 72% of cases (25,916/35,649) compared with only 5% (12,796/262,813) for occasional users (80%; 262,813/324,649). On average, power users were slightly older, more likely to have friends also using the app, and more likely to take advantage of customization features. This indicates that more engaged users are more likely to achieve positive outcomes, something that we investigate in this study.

Goyal et al [12] investigated the uptake of an app for heart disease prevention. They found that from their population of users, just 10% (5259/52,431) showed “high engagement” as measured in the number of completed in-app challenges with 85% (44,537/52,431) classed as low or very low engagers.

In terms of engagement periods, a study of usage of an app for drug adherence showed that 27% (3209/11688) used the app for at least 84 days [13]. At 165 days, 15% (82/565) of users aged above 50 years were still using the app compared with 9% (46/530) of those aged below 50 years. After a year, only 1% (6/530) of users were still engaged.

The primary focus of previous studies presented here was to consider the characteristics of longitudinal engagement and use this to gain an understanding of the user groups along with measuring usage of different features as an attribute. These studies serve as useful reference points in validating the metrics that we aimed to employ in our analysis.

### Design Philosophy and Features of the App

SAM’s design was predicated on the observation that users’ relationships with mobile devices can be an analog for aspects of face-to-face psychotherapy [14]. During development, a human-centered design process was followed with students with self-reported anxiety giving input on features and testing early prototypes [8].

In terms of usages modalities, the app was designed with flexible pathways of navigation so that users could choose to engage either in organic or more structured processes of self-help for anxiety management. This is in line with the “snowflake” model of cognitive-behavioral therapy and “reciprocal interaction” model which empowers patients to manage their own condition [15,16].

Self-monitoring is a core skill in effective self-help [17-19] and SAM provides a function to self-report on 4 dimensions of anxiety (feelings, thoughts, physiological reactions, and avoidance) and to report trends in these dimensions over time.

The app was intended to help people with moderate levels of anxiety to learn to manage that anxiety and to this end, SAM offers users a range of self-help options categorized by modality, level of challenge, and media format. This was to provide an opportunity for users to experiment and determine what works best for them [20].

Given the potential value of mobile peer connection for informational and emotional support [21,22], SAM includes a social forum—the Social Cloud—which users can join to (pseudonymously) share support and advice while learning to manage anxiety.

### Study Aims

Our enquiries in this study were therefore organized around the following core components of SAM that can be used to assess behavior: user engagement with the app, experience of anxiety as self-reported, user stated context for anxiety, use of self-help options, and peer support. In summary, our aims were as follows:

1. *User engagement with the app and user profiles over time*: To quantify engagement in terms of behavioral signatures and to characterize the user base into behavioral personas through users’ interactions with different app features
2. *Self-reported experience of anxiety*: To establish the nature and extent of self-monitoring activity by users; to understand the perceived relationships among our dimensions of anxiety used in self-monitoring; to investigate whether engagement with SAM was associated with a meaningful reduction in users’ self-reported levels of anxiety
3. *User stated context for anxiety*: To survey events and situations that users associate with anxiety and which are therefore potential foci for self-help actions
4. *Use of self-help options*: To determine whether user choice and ratings of options indicate any preferences for specific options
5. *Peer support*: To assess the extent of peer support within this community and identify gradations in the amount of support between different Social Cloud users

## Methods

### Ethics and Data Protection

Ethical approval for this project was granted by the University of the West of England, Bristol, Research Ethics Committee, Faculty of Health and Applied Sciences, Reference No. HAS.16.07.177. Use of anonymized data from the app for academic research purposes is allowed under the app’s terms of service [23].

### Dataset

The data were a snapshot of application program interface (API; cloud-based) data for the app taken in January 2017 and covering the period from July 2013 to January 2017. This included data from the activity of 105,380 registered users. Because registration with the cloud services is not mandatory in the app, this represents an estimated 15% of the total user base (based on total downloads and allowing for some redownloads by the same users).

### Data Analysis

#### Engagement Coding

Patterns of user engagement were informed by user data on anxiety monitoring, ratings of self-help options, and Social Cloud activity.

To facilitate analysis, app users were coded into binary categories according to their engagement levels. The criteria used are given in Table 1. For the interaction measures (“Significant” Posters or Monitors), the 20 updates threshold selected approximated the 95th percentile of nonzero user values. These interaction definitions were also supported by similar work (eg, [12] for a definition of “high engagement” of over 22 interactions). For the temporal measures (“long-term” monitors and posters), 14 days approximated the median of the nonzero user values.

#### Multiple Correspondence Analysis and Clustering

Given the set of engagement variables above, we wanted to see which best explained the differences between users. Taking a random sample of 10,000 users, multiple correspondence analysis (MCA) was conducted on the binary engagement variables to elicit key dimensions of variance. Because the hierarchical clustering algorithm used (Hierarchical clustering on principle components) requires the computation of a massive distance matrix, a subsample was used for computing manageability and efficiency as practiced in similar work with large datasets [11]. Rerunning the analysis with a different sample of 10,000 resulted in similar dimensions with eigenvalue variance of +/−0.01 and percent variance of +/−1.5%.

The results from MCA were used to run the cluster analysis, which was run iteratively, and suggested 4 categories of user engagement.

#### Anxiety Monitoring

Users’ experience of anxiety was derived from their self-reports of anxiety on the anxiety monitoring facility (“How’s my anxiety right now?”). Data from the 4 dimensions used, Feelings of anxiety and tension, Worrying thoughts, Avoiding things I fear, and Unpleasant physical sensations, were rated on a 0-10 scale and stored along with a timestamp for the record. We used this to derive users’ monitoring timelines and then for aggregating multiple timelines to visualize mean changes over time.

The minimum clinically important difference (MCID) is the minimum change in symptoms that is considered meaningful to the client. From reviews of its application to other mental health issues [24], we selected a criterion level for MCID of a 20% reduction in anxiety ratings, parameters as defined in Table 1, above for the “Anxiety Reducer” group.

**Table 1:** Behavior categories of users according to engagement with activity areas.

Variable	Criteria
Frequent monitor?	True if a person has recorded anxiety levels an average of once a day or more frequently through the “How’s my anxiety right now?” feature
Significant monitor?	True if a person has recorded at least 20 updates of anxiety levels
Long-term monitor?	True if a person’s anxiety tracking has spanned 14 days or more
Technique rater?	True if a person has rated a self-help technique, otherwise false
Social poster?	True if a person has ever posted to the “Social Cloud” forum
Significant social poster?	True if a person has posted at least 20 times to the “Social Cloud” forum
Anxiety reducer?	True if there was a reduction of at least 20% between the mean of the first 5 and last 5 anxiety tracking updates on 0-10 scales (mean of the “feelings of anxiety” and “worrying thoughts” scales)
Long-term social poster?	True if a person’s “Social Cloud” posts span at least 14 days

#### Anxiety Causes and Triggers

The “Things that make me anxious” feature of the app enables the user to identify anxiety triggers in a short piece of text, together with associated anxiety levels. This was used for automated content analysis.

#### Peer Support

We analyzed Social Cloud posts in terms of number of replies received, filtering to remove self-replies and extracting a complete years’ worth of data (2016). Next, to investigate the profiles of users who reply to other posts, we enumerated the number of distinct users that people had replied to.

## Results

As described in the dataset section above, we analyzed results from 105,380 registered users for whom data were logged via the app’s cloud services. Results use this entire dataset unless otherwise specified.

### User Engagement and User Profile Subgroups

Table 2 summarizes engagement levels for each of our behavioral variables. We divided anxiety monitoring into 3 variables relating to the duration and frequency of logging. Only 5% (5822/105,380) of the users were found to log anxiety levels more than once a day on average and only 2.5% (2721/105,380) made 20 or more monitoring logs and 14.9% (15,713/105,380) monitored for at least 14 days.

According to our stringent MCID defined above, 2.2% (2327/105,380) of the user base could be said to be anxiety reducers.

In terms of the rating of self-help techniques, 5.5% (5,862/105,380) submitted at least one rating. On the Social Cloud functions, 25.6% (2781/105,380) posted at some point in their usage of the app with only 0.4% (522/105,380) posting 20 or more times and 3.7% (3973/105,380) posting over an extended period.

Our dimensionality computation using MCA on these behavioral variables gave the 7 dimensions shown in Table 3. The first 2 dimensions explained 50% of the variance and the first 5 explained 85%. The relatively low initial 2 eigenvalues and percentage variance explained indicate that the dimension reduction is only partially successful and is explained by the large dataset and the fact that only small percentages of users show “extreme” specialized engagement and a larger percentage show more moderate and mixed engagement.

**Figure 1:**
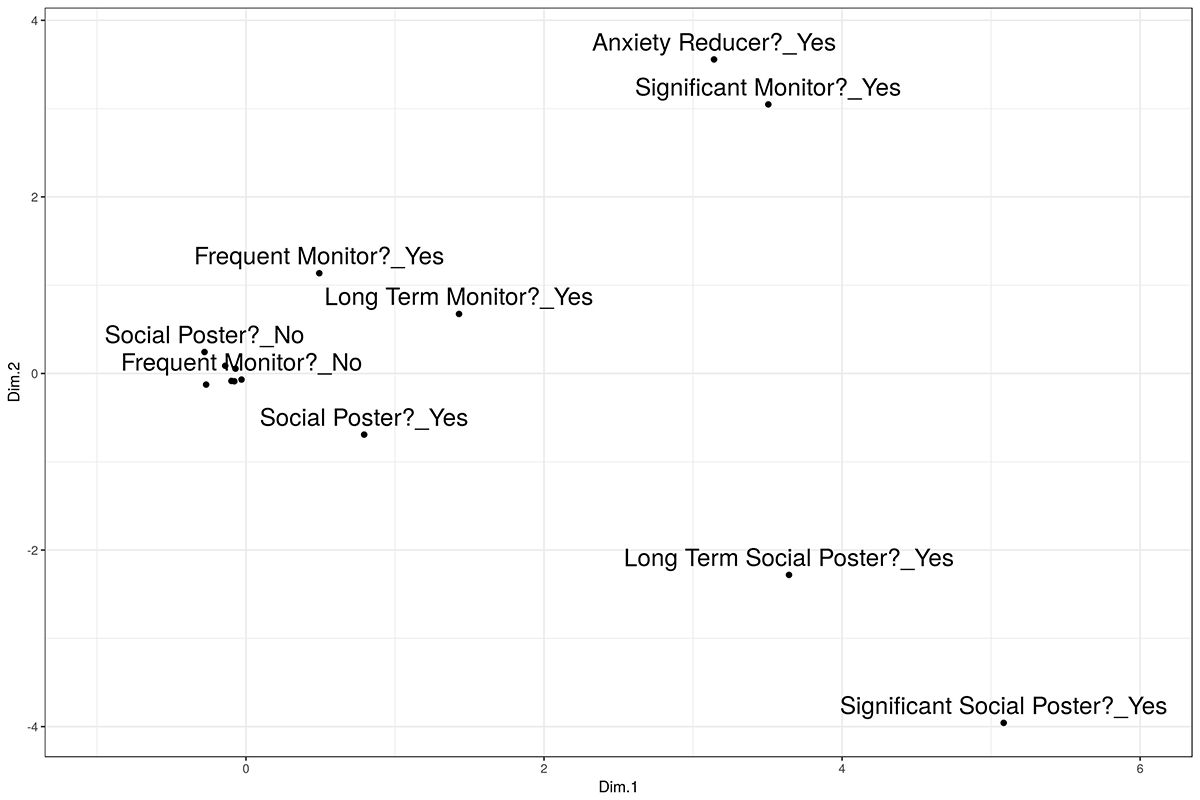
Multiple correspondence analysis-variable map. Dim: dimension.

Figure 1 illustrates the variable values against the first 2 MCA dimensions, indicating a bifurcation at the more extreme ends by social engagement and monitoring engagement. Anxiety reducers were most closely correlated with significant monitors. Long-term social posters also posted significant levels of content.

**Figure 2:**
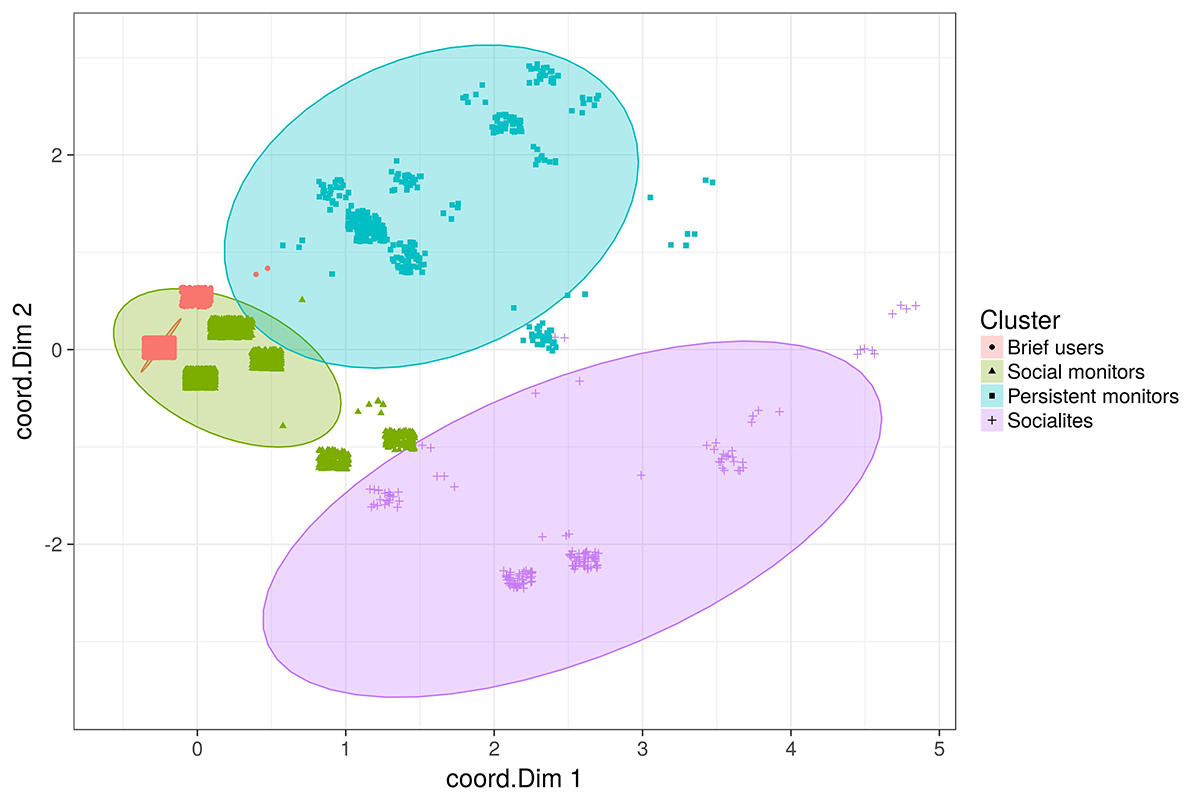
Cluster analysis: groups (jittering added to points, ellipses show 95% normal confidences). coord.Dim: dimension coordinates.

Figure 2 illustrates the cluster membership against the 2 principle MCA dimensions. Multimedia Appendix 1 shows the cluster statistics for each variable value.

**Table 2:** Engagement levels by activity (N=105,380).

Variable	Users that answered yes, n (%)
Frequent monitor?	5,822 (5.52)
Significant monitor?	2,721 (2.58)
Long-term monitor?	15,713 (14.91)
Anxiety reducer?	2,327 (2.21)
Technique rater?	5,862 (5.56)
Social poster?	27,081 (25.70)
Significant social poster?	522 (0.50)
Long-term social poster?	3,973 (3.77)

**Table 3:** Components with percentage of variance explained.

Multiple correspondence analysis dimension	Eigenvalue	Percentage of variance	Cumulative percentage of variance
1	0.30	29.54	29.54
2	0.21	20.55	50.09
3	0.14	14.24	64.33
4	0.11	11.48	75.80
5	0.09	9.29	85.09
6	0.08	8.24	93.33
7	0.07	6.67	100.00

**Table 4:** Summary of anxiety monitoring by users (N=105,380).

Anxiety monitoring	Number of users, n (%)
At least once	50,509 (47.93)
More than one day	27,951 (26.52)
14 days or more	15,713 (14.91)
30 days or more than 9 times	4909 (4.65)
20 times or more	2721 (2.58)

The 4 cluster categories appear to have the following characteristics supported by the data in Multimedia Appendix 1 (see Multimedia Appendix 1 for group membership and statistics from the MCA output, as defined in [25]).

- *Cluster 1: Brief Users* (65% of sample) monitor their anxiety more than once a day for a short period of time. They do not post on the Social Cloud.
- *Cluster 2: Social Monitors* (30% of sample) are defined by low levels of anxiety monitoring and Social Cloud posts over a longer period of time.
- *Cluster 3: Persistent Monitors* (3.5%) engage in high levels of anxiety monitoring over time with a low level of posts on the Social Cloud.
- *Cluster 4: Socialites* (1.5%) are defined by a high level of Social Cloud posts over time with low levels of anxiety monitoring.

### Experience of Anxiety

In terms of self-monitoring using the “How’s my anxiety” app feature, Table 4 summarizes their use of this facility. Out of our 105,380 users, less than half monitored their anxiety at least once and only 2.5% monitored it 20 times or more.

Correlations between the anxiety monitoring dimensions are shown in Table 5. All correlations were significant at *P*<.001. There were moderate to high correlations (0.4 to 0.7) between each of the self-rated dimensions of user anxiety. Feelings of anxiety and tension were most strongly associated with both worrying thoughts and unpleasant physical feelings. Avoidance was most strongly associated with worrying thoughts and least strongly with unpleasant physical sensations. Although there was some differentiation, the equivalence of correlations might indicate that some users were not discriminating between the 4 components of anxiety.

#### Change in Levels of Anxiety

**Figure 3:**
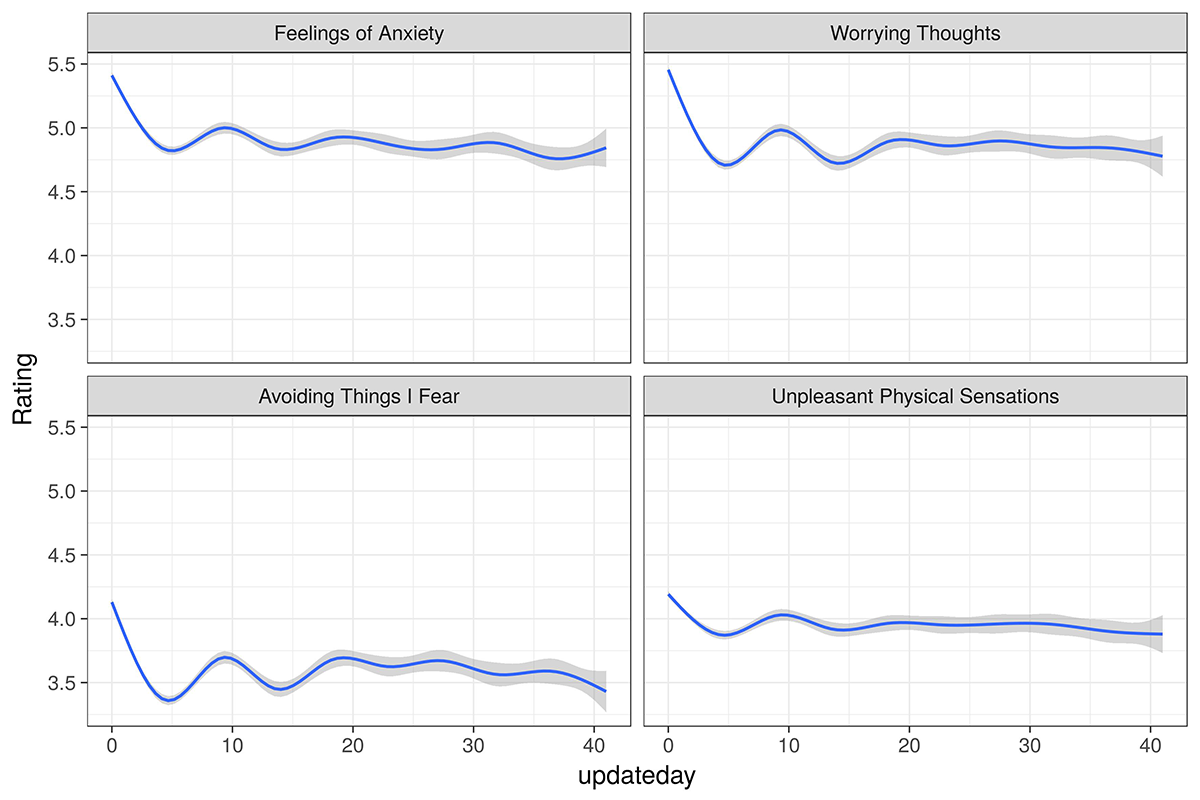
Anxiety levels over the first 6 weeks of monitoring (235, 286 observations with generalized additive model-GAM-smoothing), 95% CI shading. Ratings on 0-10 scale where 10 is highest. Updateday is the days elapsed since the user began monitoring.

Anxiety monitoring over time is shown in Figure 3. This covers the first 6 weeks of monitoring by all monitoring users.

The graphs in Figure 3 show a downward trend on all 4 dimensions of anxiety over the measurement period of 40 days. There is a marked dip in aggregated mean anxiety within the first 5 days of using the app; following that, there is variability in the mean anxiety levels across all 4 dimensions with no return to the initial level of anxiety.

#### Meaningful Change

**Figure 4:**
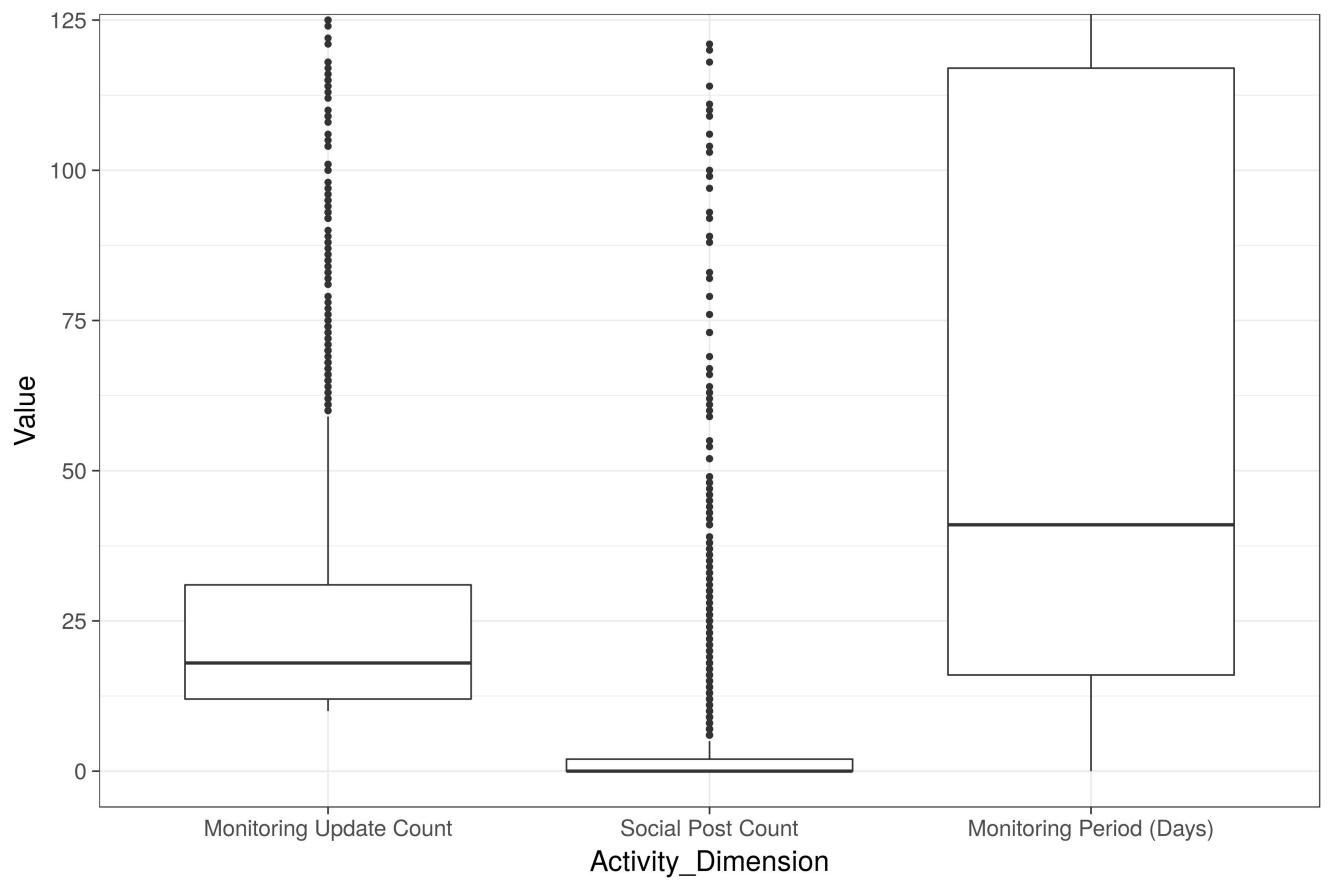
Activity summary for the "anxiety reducers" group (N=2327). Boxes and whiskers show quartiles with outliers as individual points.

Of the sample (2327 users), 2.2% met our MCID criterion for anxiety reduction. Because this group is of interest in terms of our primary outcome for the app, we also looked in more detail at the behavioral characteristics of the group. Figure 4 summarizes the monitoring and Social Cloud activity for this group. As indicated by our earlier clustering, the anxiety reducers tended not to post on the Social Cloud and typically had a relatively low monitoring count dispersed over a relatively long period of time.

### Causes of Anxiety

**Figure 5:**
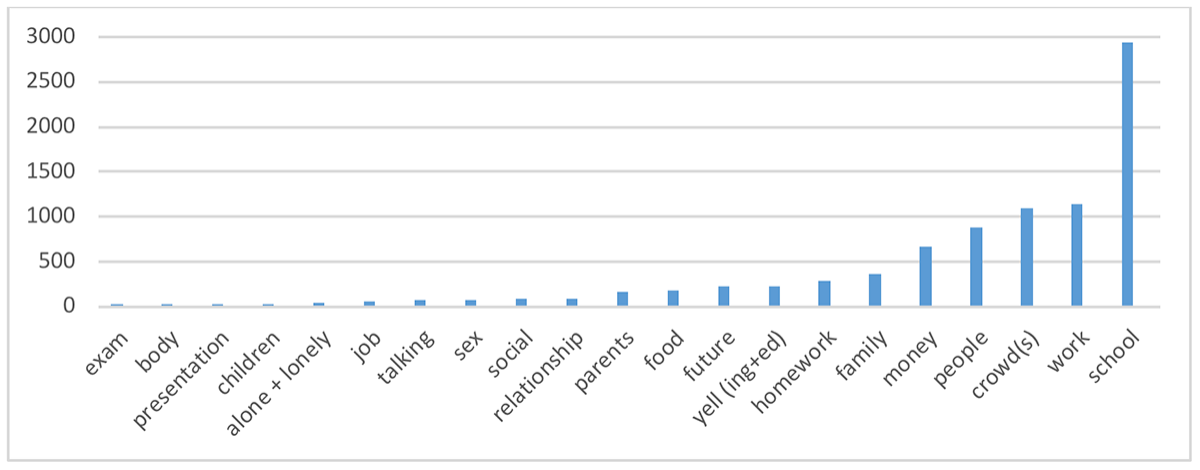
Frequency of a sample of key words occurring in "Things that make me anxious" entries.

In addition to the self-reported anxiety levels, we also investigated self-reported triggers and causes. In total, there were 105,898 triggers recorded by 35,700 (33.88%) of the registered users with 6072 (5.76%) users making 5 or more entries. The frequency of occurrence of a sample of significant key words is shown in Figure 5.

**Figure 6:**
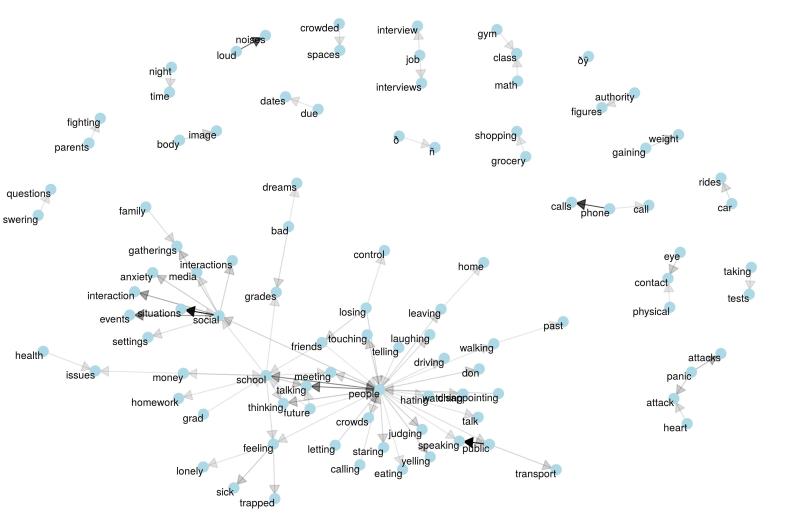
Network of bigram associations for most common bigrams in user anxiety causes (bigrams with 30 or more occurrences).

The corpus of anxiety triggers was further analyzed for common bigrams (2-word phrases) and an association graph between these was constructed, as seen in Figure 6.

### Use of Self-Help Options

We aggregated the ratings that had been made for the self-help techniques across all users. Table 6 shows the ordering of the most popular self-help options by mean user ratings, showing the number of times each was rated and the self-help category to which each technique belongs. We find highly rated (> 4/5 star) techniques across all of our content categories though, as noted below, the most frequently-rated techniques were associated with the quick-access “Help for anxiety now” screen. We note that mental and motivational information and techniques were among the most highly rated, gaining an average of 4.2 out of 5 and above.

**Table 5:** Cross-correlation of users’ self-ratings on 4 dimensions of anxiety. (N=361,246 updates by 55,479 distinct users).

Dimension	Feelings of anxiety and tension	Worrying thoughts	Avoiding things I fear	Unpleasant physical sensations
Feelings of anxiety and tension	1	0.69	0.49	0.68
Worrying thoughts	0.69	1	0.53	0.53
Avoiding things I fear	0.49	0.53	1	0.44
Unpleasant physical sensations	0.68	0.53	0.44	1

Looking at how many techniques different users have rated, we note that most people have rated only a small number of those available (Table 7).

### Peer Support

**Figure 7:**
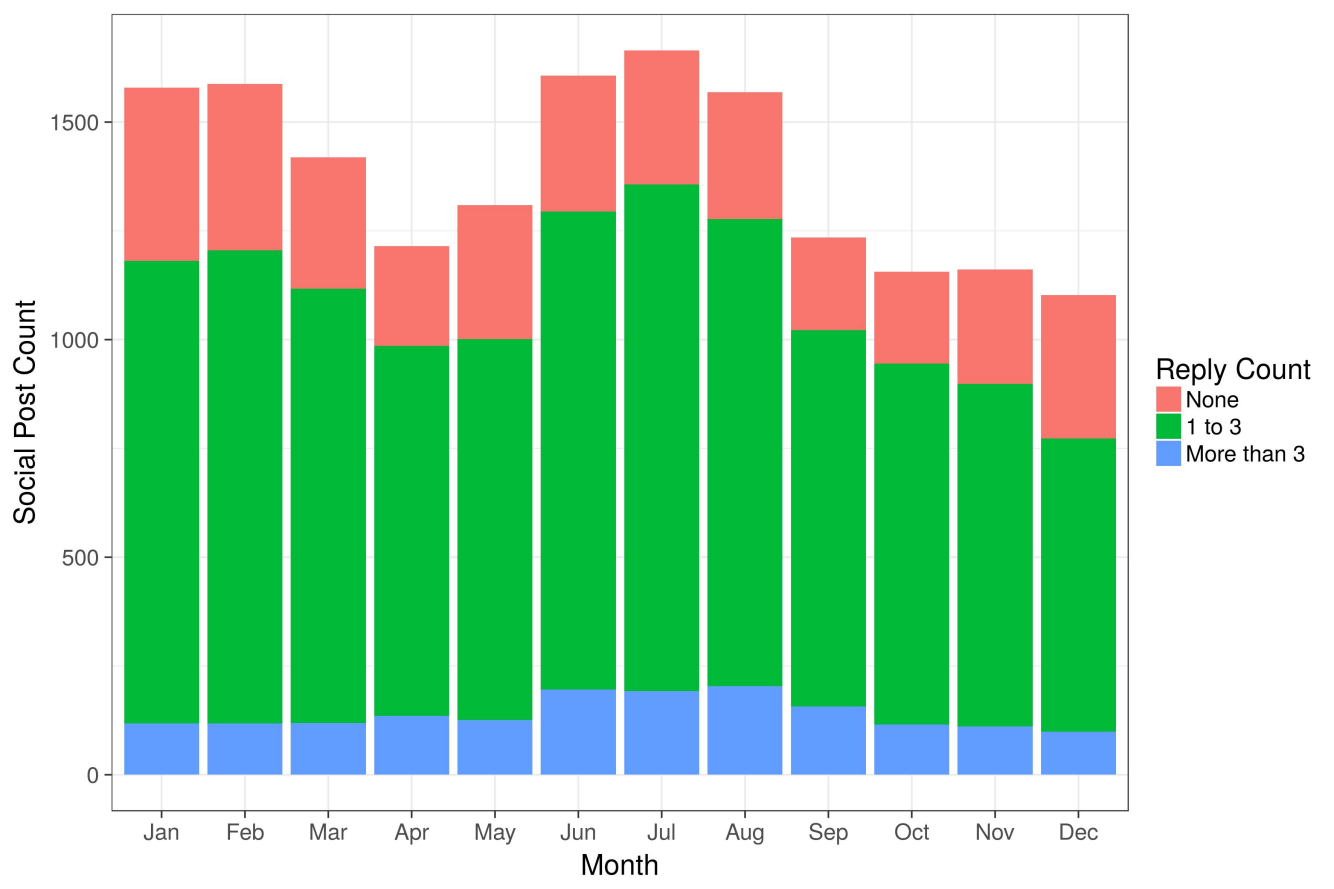
Social Cloud activity by month for 2016, showing total posts and the replies received.

Table 2 shows that one quarter of users (27,081) posted at least one message on the Social Cloud with less than 4% posting for more than 50 days. A very small group (0.4%, 522 users) posted more than 50 times. In terms of replies to social posts, we firstly observed that a large proportion of posts (in the sense of “new threads”) received at least one reply from another app user, as seen in Figure 7.

**Figure 8:**
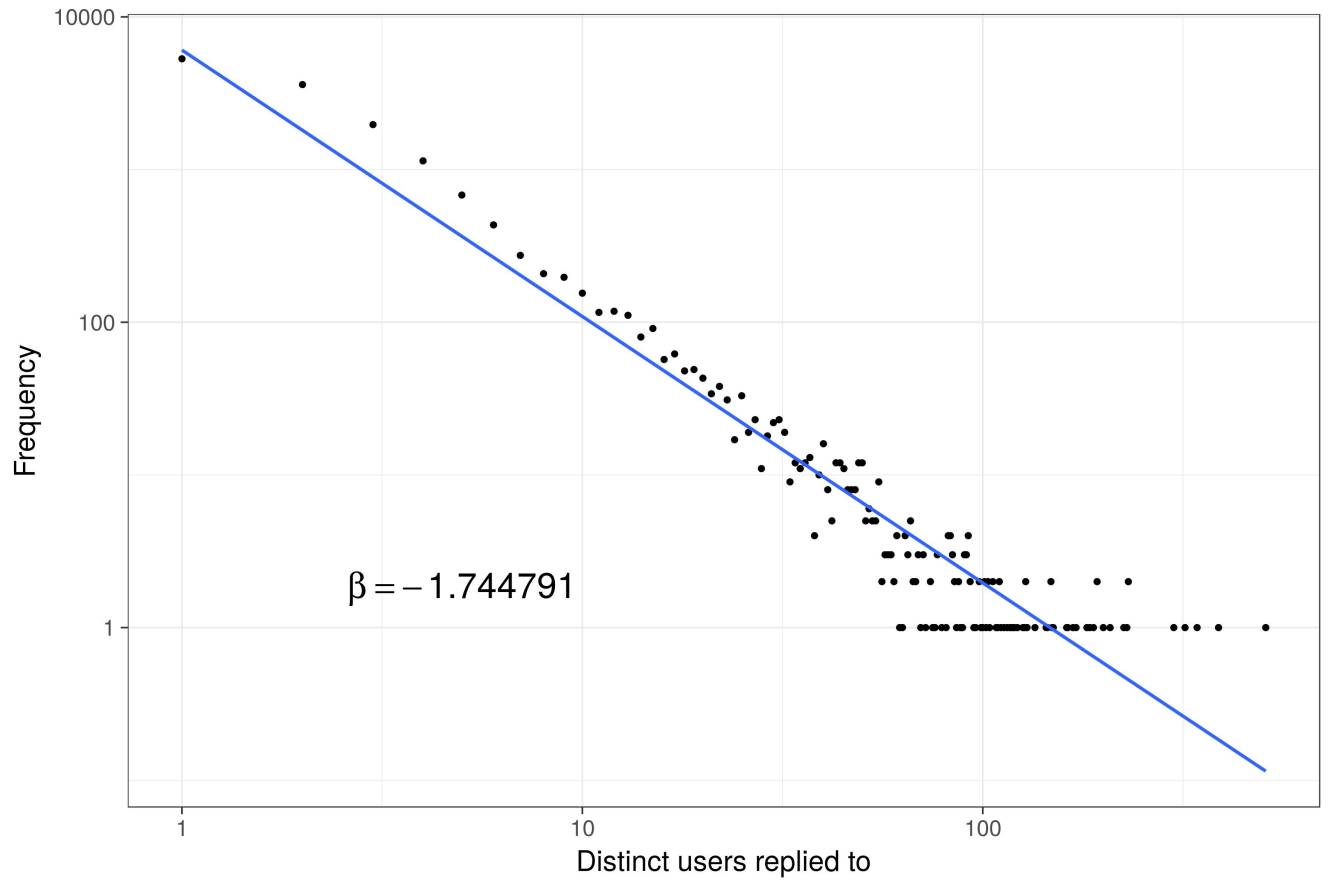
Extent of replying to others' posts (log10 scales).

In terms of who is doing the replying, when graphed on a log scale (frequency vs users replied to), we observed an approximate inverse power law trend, as seen in Figure 8, indicating that a small percentage of profiles are responsible for a very large number of Social Cloud replies.

**Table 6:** Top 12 most popular self-help options ordered by mean rating (most popular first), N=15,437 ratings by 5862 app users.

Self-help option	Description	Section	Ratings, n	Mean rating^a^
Stop that thought	A self-help intervention based on the idea that persistent worrying thoughts can be suppressed or diverted by forceful inner speech or external actions	Mental relaxation	528	4.26
You can do it	Encourages positive thinking about making changes using personal examples from survey interviews	Small steps	124	4.25
You’re biased!	Provides a digest of research-based information on how cognitive biases influence our experience of anxiety	Information	530	4.24
Examples of anxious thinking	Describes common patterns of thought derived from practice-based research in cognitive therapy	Thinking	527	4.24
Picture peace	Uses contemplation of and physical contact with selected visual images to shift attention away from anxious experience	Mental relaxation	2222	4.21
Checklist	Provides a summary reminder of the key principles of learning to manage anxiety using SAM^b^	Small steps	239	4.20
A simple meditation	Uses well-established meditation guidance to clear the conscious mind of thoughts and sensations	Mental relaxation	190	4.13
Calm breathing	Uses a well-established breathing exercise to achieve a basic level of physical and mental calm	Physical relaxation	2186	4.08
Ground yourself 2	Uses associative learning to establish links between positive memories and low arousal	Physical relaxation	231	4.06
Symptoms of anxiety	A graphic which aims to show the diversity of anxiety symptoms within 4 psycho-physical categories	Information	617	4.06
A cycle of anxiety	A graphic to show how feelings, sensations, beliefs, and behavior interact to create and maintain anxiety	Information	604	4.06
Read this twice, slowly	A self-help module whose instructions and linked content are intended to provide some immediate relief from anxiety	Help for anxiety now	467	4.03

^a^Out of 5, where 5 is highest.

^b^SAM: Self-help for Anxiety Management [app].

**Table 7:** Number of users giving ratings, by number of techniques rated (N=5862 users).

Number of options	Number of users, n (%)
0-5	5304 (90.48)
5-10	362 (6.18)
10-15	113 (1.93)
15-20	27 (0.46)
20-25	26 (0.44)
25-30	17 (0.29)
30-35	13 (0.22)

## Discussion

### Principal Findings

This study aimed to enlarge our understanding of how people use a self-help app for anxiety management. We analyzed user data on anxiety-provoking events, self-monitoring of anxiety, ratings of self-help options, and social network posts.

Our main findings, in summary, were as follows:

1. There was an inverted pyramid or “funnel” of anxiety monitoring with decreasing numbers of users making increasing use of the facility. Out of 105,380 users in our sample, fewer than half monitored their anxiety at least once and only 2.5% monitored it 20 times or more. Our persona profiling showed significantly different subgroups based on engagement levels and the extent of monitoring and social activity with very active social users tending not to monitor extensively and vice versa.
2. Anxiety monitoring by all users showed an initial dip in mean aggregated anxiety ratings over the first few days of use, followed by a more mixed profile subsequently. There was a partial correlation between our anxiety component scales.
3. Anxiety triggers were varied but centered on those more associated with early adolescence.
4. The most highly rated self-help options were those associated with our “Help for anxiety now” screen, though physical relaxation and informational options also featured highly in the ratings.
5. Activity on the Social Cloud showed a similar funneling to monitoring with only one quarter of registered users posting at least one message, less than 4% posting for more than 50 days, and a very small group (0.4%) posting more than 50 times. A similarly small group contributed to a large amount of the social support through providing replies to others’ posts.

We feel that findings are consistent with stage-based models of help seeking (eg, [26]) and with a consumer choice ethos where consumers and clients felt entitled to explore and evaluate their health care options. Exploration of its functions helps people to decide whether they wish to persist with a particular self-help device. It is these “visitors” to SAM who populate the top layer of the inverted pyramid. We will now explore the findings in these different areas in more detail.

Engagement patterns were overall similar to those noted in other mHealth user populations [11,12] with a high number of low engagers and a long tail of more active users. The attributes of our 4 clusters (Brief Users, Social Monitors, Persistent Monitors, and Socialites) offered some clues to user engagement with SAM.

There are help-seeking preferences in that Socialites value social sharing, whereas Persistent Monitors prefer tasks such as self-monitoring and self-help activities. Further work is needed to understand if these differences have a link to gender, as suggested by Pedersen [27]. Certainly, systematic reviews and phased models (eg, [28-33]) provide evidence that matching therapy to client preferences has a positive impact on therapeutic engagement and therapy outcomes.

For anxiety reduction, the graphical summaries of anxiety monitoring showed a downward trend on all 4 dimensions of anxiety over the measurement period of 40 days. There was a marked dip in anxiety within the first 10 days of using the app; following that, there was variation in anxiety levels across all 4 dimensions with no return to the initial level of anxiety. The data were consistent with several perspectives on personal development.

Frank and Frank [34] proposed that people seek help because they are demoralized by being unable to manage their problems. Contacting a source of help instilled hope that change could occur and reduced anxiety. We have suggested previously [8] that “common factors” in psychotherapy [35,36] such as hope, credibility, and autonomy might also apply to digital mental health devices including a self-help app. If true, one would expect some reduction in anxiety in the initial period of anxiety monitoring.

Studies of change without professional help, for example [37], indicated that change is a gradual process, sometimes emotionally turbulent, and takes months rather than weeks. For those receiving psychotherapy, initial severity of symptoms, individual differences, and the ongoing challenges of emotion regulation contribute to variations in the pattern of change [38]. A recent large-scale study of counseling clients with varying levels of psychological well-being [39] also indicated that trajectories of change are diverse. Researchers on the dose-effect relationship in psychotherapy concur that around 50% of patients are measurably improved after 8 weekly sessions, a treatment period of 56 days [40,41]. Thus, our users’ 40-day monitoring period may be a small but revealing slice of a longer process.

Only a small percentage of users achieved a criterion reduction in anxiety. With medians of 18 anxiety updates and 41 days anxiety monitoring (upper quartile over 100 days), reductions in anxiety were associated with sustained anxiety monitoring. They were not associated with Social Cloud activity where the median number of posts was zero. Whatever external support the Social Cloud users in the other clusters received, it was not associated with criterion reductions in reported anxiety.

While noting that this study did not exclude people who might concurrently have been enrolled in a therapeutic program or intervention, this group might indicate a larger population of users learning to manage their anxiety; if the percentages are scaled up for 1 million downloads (SAM’s approximate uptake at September 2017), there would be 37,300 users. This is similar in magnitude to 10.7% of the 346,412 annual referrals to United Kingdom’s National Health Service Improving Access to Psychological Therapies service for anxiety or stress-related disorders (p24, Figure 10) [42].

For causes of anxiety, Figures 5 and 6 indicate the main areas of anxiety for the user sample. There are many references to social relations—people, talk, meeting, and touching. They include evaluative aspects of those relations such as public speaking, watching, judging, and hating. The situations described and other references suggested a user group in the adolescent to young adult stage of development, including school, parents, authority, class, grades, and interviews. With a user group in middle to late adulthood, one would expect more references to jobs and careers, family and children, and finances and health care contexts [43]. Some of these midlife anxieties were noticeably limited in this user group.

We also looked at user of self-help options. The app was designed to offer a range of self-help options for anxiety management, differentiated by presentation mode and psycho-educational focus. The fact that all 34 options were given ratings suggests that each was potentially meeting a need for some portion of the user sample.

Although user ratings indicated a moderate to high level of satisfaction with the self-help options, they were provided by only 5.5% of the sample and over 90% of those users gave ratings to no more than 5 out of 34 available options. Users were encouraged to explore the range of self-help options and it was assumed that they may not rate them unless engaged with them over time.

Based on the frequency of user ratings, 3 of the top 5 options were “Calm Breathing,” “Picture Peace,” and “Change the Focus.” These were accessed from the "Help for anxiety now" module which is intentionally prominent on the app’s main menu page; it is likely that these options for managing immediate anxiety or panic will attract users. All 4 of the “Information about Anxiety” options featured in the top 10 of the frequency list and 2 of them in the top 5 with the "Help for anxiety now" options. In contrast, 6 of the 8 options in the “Making Changes” module featured in the bottom 10 of the frequency list.

These rankings suggest that actions to contain immediate anxiety with information and directed self-help are primary uses of the app and are preferred over sustained self-help activity involving a range of options. They support the view of many users being in the early stages of commitment to a personal change process, as outlined above.

A complementary view is that SAM is being used to provide what users expect apps to provide. In a content analysis of app store descriptions, the most commonly stated purpose of apps was symptom relief and information about mental health; the most frequently mentioned self-help options were those for mild anxiety, such as relaxation [44].

From an interaction design perspective, these ratings provide excellent evidence for future iterations of the self-help techniques and the addition of new tools into the app. In this way, the available self-help techniques might be allowed to evolve based on user preferences.

As far as social peer support is concerned, three quarters of the users who registered for the Social Cloud did not take part in its interactions but may have nonetheless logged in to absorb the views and experiences of others. For them and for those users who were more socially active, there are therapeutic factors in group psychotherapy which may apply in Web-based forums [45], such as learning that others have similar concerns, raising hope that things can change, and gaining information that is helpful in dealing with personal concerns. These factors are supported by recent studies of Web-based support which have identified information exchange, sharing experiences, emotional support, and encouragement as the most common interactions [46,47].

Overall, the low levels of sustained engagement with the Cloud in our sample (less than 5%) and the number of registrations as a proportion of total downloads (15%) indicate that the Social Cloud appeals to a subset of users rather than to the majority of those who download the app.

User attributions for causes of their anxiety (above) suggest an adolescent or early adulthood user group, a developmental period which is associated with higher levels of social-evaluative anxieties. The limited appeal of the Social Cloud tends to support that view. Being anxious may be experienced as shameful [48], and this will discourage social sharing.

### Limitations

The dataset was based on a sample of users who downloaded SAM and also registered with its Social Cloud. Our findings may not apply to the greater proportion that downloaded but did not register.

The research quoted on the duration and trajectories of change in personal development processes indicates that from a large dataset covering a short period of user activity, we should be cautious in our generalizations about patterns of engagement and change.

Where the findings are based on self-reporting by users, as in monitoring of anxiety levels or rating self-help options, there was no standard guidance for users on how to make those assessments. In this absence, self-monitoring of anxiety will be guided by subjective criteria and individual baselines. Further research should aim to confirm reductions in anxiety using a validated measure of anxiety.

Our reflections on the statistical analyses, hypothesizing links between user behavior and psychological processes, could not be contextualized by qualitative data from users. The value and meaning of the user experience with SAM remains a matter for further investigation.

### Conclusions

The analysis suggests a scenario of initial downloads by a large body of prospective users, followed by successive withdrawals from engagement, leaving a small core of committed and effective users—an inverted pyramid of engagement. Within this process of narrowing engagement, there are clusters of users, notably those focused differently on the self-monitoring and peer support functions of the app.

Causal attributions for anxiety suggest a user group in adolescence and early adulthood who have particular anxieties about self and social relations. The indications from rating and frequency data on the app’s self-help options indicate that help for immediate anxiety might be a primary motive for using the app.

Anxiety reduction is most associated with persistence in self-monitoring and we might assume that those users are similarly diligent in their use of self-help options; a review by Newman et al [20] concluded that self-help interventions for anxiety are most effective with motivated users.

### Recommendations

Our analyses of users’ patterns of engagement with the app as presented here will be of value to other mHealth apps offering self-help for common mental health concerns. Reflections on these patterns will inform practitioners seeking to engage with clients using self-help apps. Service managers will need to take account of how client populations respond to mHealth opportunities to promote them appropriately. App developers may wish to consider how engagement can best be supported through in-app guidance and external prompts. They will need to work closely with practitioners to increase the validity of self-monitoring and rating systems and consider how a more guided usage approach might be built into the app as an implicit aspect of its design.

Suler [49] has researched and written extensively about how people use social media, their forms of engagement, and the interaction between personality types and Web-based engagement. He is clear that the architecture of Web-based life offers many routes to personal development; media references such as “a therapist in your pocket” [50] applaud the immediacy and accessibility of apps without recognizing the varieties of user engagement shown in this study.

User motivation and personalization of therapy resources are critical to engagement with the therapeutic program [51]. We propose that there is a task for therapeutic practitioners and organizations who wish to promote digital mental health, that is, matching the digital support to the individual user with regard to patterns of and preferences for mobile engagement as they would in face-to-face therapy. For autonomous self-help by large, diverse user populations, this will mean comprehensive in-app guidance, links to Web-based support in a range of formats, and options for integrating mobile self-help with offline therapy.

Practitioners working with app users will need to adopt a flexible role in matching therapeutic needs to digital options. They can offer encouragement for persistence with autonomous self-help activities; be active in helping their clients make best use of their apps; and collaborate to select self-help options in support of a program of face-to-face therapy. There is a parallel with art therapy where interaction between client, therapist, and image is employed to facilitate personal understanding and options for change [52]. Practitioners will want to consider the benefits and the challenges of their clients and users having attachments to, and communications between, both person and digital device.

## Appendix

Multimedia Appendix 1 Characteristics of user clusters.

## Abbreviations

MCA: multiple correspondence analysis
MCID: minimum clinically important difference
SAM: Self-help for Anxiety Management
