# Supplementary material for: Interaction and Engagement with an Anxiety Management App: Analysis Using Large-Scale Behavioral Data
Source: JMIR Ment Health. 2018 Oct 1;5(4):e58. doi: 10.2196/mental.9235 (PMC6324647; doi:10.2196/mental.9235)
Supplement: Multimedia Appendix 1 [file mental_v5i4e58_app1.pdf]

Multimedia Appendix

Table 9: Cluster 1, Brief Users (N=*6465* of *10000* sample)

	Cla/Mod	Mod/Cla	Global	P.value	v.test
Social Poster? = No	86.5	100.0	74.7	0	Inf
Long Term Monitor? = No	76.5	100.0	84.5	0	Inf
Long Term Social Poster? = No	67.3	100.0	96.0	0	29.1
Significant Monitor? = No	66.5	100.0	97.3	0	24.0
Anxiety Reducer? = No	66.2	100.0	97.6	0	22.4
Significant Social Poster? = No	65.7	100.0	98.5	0	17.8
Frequent Monitor? = No	65.2	95.2	94.4	0	4.8
Frequent Monitor? = Yes	55.1	4.8	5.6	0	-4.8
Significant Social Poster? = Yes	0.0	0.0	1.5	0	-17.8
Anxiety Reducer? = Yes	0.0	0.0	2.4	0	-22.4
Significant Monitor? = Yes	0.0	0.0	2.7	0	-24.0
Long Term Social Poster? = Yes	0.0	0.0	4.0	0	-29.1
Social Poster? = Yes	0.0	0.0	25.3	0	-Inf
Long Term Monitor? = Yes	0.1	0.0	15.5	0	-Inf

Notes on Table:
Cla/Mod = Percent of users with that variable value who are in this cluster
Mod/Cla = Percent of users in this cluster who have that variable value
Global = Percent of all users with the variable value
P.value = Significance of v-test statistic
v.test = A test of deviation from the population mean. Magnitude and sign indicates over- or under-representedness in current cluster
[see 52]

Table 10: Cluster 2, Social Monitors (N=*3009* of *10000* sample)

	Cla/Mod	Mod/Cla	Global	p.value	v.test
Social Poster? = Yes	89.1	74.9	25.3	0	Inf
Long Term Monitor? = Yes	73.8	38.0	15.5	0	Inf
Significant Monitor? = No	30.9	100.0	97.3	0	13.9
Anxiety Reducer? = No	30.8	100.0	97.6	0	13.0
Long Term Social Poster? = Yes	59.3	7.8	4.0	0	12.3

	Cla/Mod	Mod/Cla	Global	p.value	v.test
Significant Social Poster? = No	30.6	100.0	98.5	0	10.3
Frequent Monitor? = No	30.5	95.7	94.4	0	4.0
Frequent Monitor? = Yes	22.7	4.3	5.6	0	-4.0
Significant Social Poster? = Yes	0.0	0.0	1.5	0	-10.3
Long Term Social Poster? = No	28.9	92.2	96.0	0	-12.3
Anxiety Reducer? = Yes	0.0	0.0	2.4	0	-13.0
Significant Monitor? = Yes	0.0	0.0	2.7	0	-13.9
Social Poster? = No	10.1	25.1	74.7	0	-Inf
Long Term Monitor? = No	22.1	62.0	84.5	0	-Inf

Table 11: Cluster 3, Persistent monitors (N=373 of 10000 sample)

	Cla/Mod	Mod/Cla	Global	p.value	v.test
Significant Monitor? = Yes	90.8	66.5	2.7	0	Inf
Anxiety Reducer? = Yes	91.7	59.0	2.4	0	38.3
Long Term Monitor? = Yes	20.9	86.9	15.5	0	31.5
Frequent Monitor? = Yes	19.5	29.5	5.6	0	15.1
Long Term Social Poster? = Yes	9.3	9.9	4.0	0	5.1
Social Poster? = Yes	4.9	33.0	25.3	0	3.4
Significant Social Poster? = No	3.8	100.0	98.5	0	3.0
Significant Social Poster? = Yes	0.0	0.0	1.5	0	-3.0
Social Poster? = No	3.3	67.0	74.7	0	-3.4
Long Term Social Poster? = No	3.5	90.1	96.0	0	-5.1
Frequent Monitor? = No	2.8	70.5	94.4	0	-15.1
Long Term Monitor? = No	0.6	13.1	84.5	0	-31.5
Anxiety Reducer? = No	1.6	41.0	97.6	0	-38.3
Significant Monitor? = No	1.3	33.5	97.3	0	-In

Table 12: Socialites (N=153 of *10000*)

	Cla/Mod	Mod/Cla	Global	p.value	v.test
Significant Social Poster? = Yes	100.0	100.0	1.5	0	Inf
Long Term Social Poster? = Yes	31.3	81.0	4.0	0	26.3

	Cla/Mod	Mod/Cla	Global	p.value	v.test
Social Poster? = Yes	6.0	100.0	25.3	0	20.5
Long Term Monitor? = Yes	5.2	52.3	15.5	0	10.6
Significant Monitor? = Yes	9.2	16.3	2.7	0	7.2
Anxiety Reducer? = Yes	8.3	13.1	2.4	0	6.2
Frequent Monitor? = Yes	2.7	9.8	5.6	0	2.1
Frequent Monitor? = No	1.5	90.2	94.4	0	-2.1
Anxiety Reducer? = No	1.4	86.9	97.6	0	-6.2
Significant Monitor? = No	1.3	83.7	97.3	0	-7.2
Long Term Monitor? = No	0.9	47.7	84.5	0	-10.6
Social Poster? = No	0.0	0.0	74.7	0	-20.5
Long Term Social Poster? = No	0.3	19.0	96.0	0	-26.3
Significant Social Poster? = No	0.0	0.0	98.5	0	-Inf

Abbreviations

HFAN: Help for anxiety now
MCA: Multiple correspondence analysis
MCID: Minimum clinically important difference
UK: United Kingdom
